# On Medical Degrees

**Published:** 1802-02

**Authors:** Philo Medicus

**Affiliations:** Scotland


### *On Medical Degrees.*

[ Continued from our laſt, p. 13. ]

IF no radical cure can be obtained for this abuſe, might it not be proper to add a diſtinguiſhing mark to ſuch Phyſicians? Let them be pointed out as *impoſtors* of a *double dye.* An impoſtor who claims a title at his own hand may, perhaps, be excuſed; but the men who *falsley* obtain, and thoſe who *falsley* confer the ſanctions of a Univerſity, are certainly the moſt abandoned. The degree of a Univerſity ſhould be ſacred to merit. When a man aſſumes the title of Phyſician, the public naturally confide in his knowledge and abilities: but when to this, he publiſhes the ſanctions of a Univerſity, their faith becomes ſtronger. What muſt be the reſult of this confidence in the caſe of a man deſtitute of knowledge? It is like committing the charge of a ſhip, in the moſt critical ſituation, to a man who has never handled a rudder, nor ſeen a compaſs.

When Dr. Johnſon was at St. A******, in his tour to the North of Scotland, he was frequently ſpeaking of the original ſplendour of the place. The Profeſſors and other people about the Univerſity aſſured him, that the preſent decline was not owing to any deficiency on their part, *but they were very poor.* "Never mind that," ſays the Doctor, "you will get rich by DEGREES." This is now literally the caſe; for ſince the ſcheme of publiſhing a liſt of all the phyſicians took place, the applications have been very frequent, and the conferers of theſe honours are actually flattering themſelves that they ſhall get "Rich by Degrees." Men, who could not even be admitted to public examinations, as ſurgeons, are now daſhing away with their degrees as phyſicians! If this was a matter of notoriety at the time Johnſon made his tour, it is ſtill more ſo now.

By theſe reflections I am certain to gain their ill will. But as I have now ſet out in the cauſe of the public, I am determined to go on; and if any of *theſe Phyſicians* conſider themſelves as injured by the likeneſs I have drawn of them, I ſhall be happy to hear their defence, and give them every ſatisfaction in my power. You may expect to hear further from me on this ſubject. I am, &c.

PHILO MEDICUS.

*Scotland, Dec.* 1, 1801.

P. S. In my next, I ſhall preſent you with a few portraits of our St. A****** Phyſicians.

CRITICAL

